# Gut resident *Escherichia coli* profile predicts the eighteen-month probability and antimicrobial susceptibility of urinary tract infections

**DOI:** 10.1101/2024.04.05.24305377

**Published:** 2024-04-09

**Authors:** Veronika Tchesnokova, Lydia Larson, Irina Basova, Yulia Sledneva, Debarati Choudhury, Thalia Solyanik, Jennifer Heng, Teresa Cristina Bonilla, Isaac Pasumansky, Victoria Bowers, Sophia Pham, Lawrence T. Madziwa, Erika Holden, Sara Y. Tartof, James D. Ralston, Evgeni V. Sokurenko

**Affiliations:** 1 Department of Microbiology, University of Washington School of Medicine, 1705 NE Pacific St., Seattle, WA 98195, USA; 2 Kaiser Permanente Washington, 2715 Naches Ave. SW, Renton, WA 98057, USA; 3 Kaiser Permanente Washington Health Research Institute, 1730 Minor Ave, Suite 1600, Seattle, WA 98101-1466, USA; 4 Kaiser Permanente Southern California, Department of Research & Evaluation, Pasadena, 100 S Los Robles, Pasadena, CA 91101, USA; 5 Kaiser Permanente Bernard J. Tyson School of Medicine, Department of Health Systems Science, 100 S Los Robles, Pasadena, CA 91101, USA

## Abstract

**Background:**

Community-acquired UTI is the most common bacterial infection managed in general medical practice that can lead to life-threatening outcomes. While UTIs are primarily caused by *Escherichia coli* colonizing the patient’s gut, it is unclear whether the gut resident *E. coli* profiles can predict the person’s risks for UTI and optimal antimicrobial treatments. Thus, we conducted an eighteen-month long community-based observational study of fecal *E. coli* colonization and UTI in women aged 50 years and above.

**Methods and Findings.:**

We enrolled a total of 1,804 women distributed among age groups 50–59 yo (437 participants), 60–69 yo (632), 70–79 yo (532), and above 80 yo (203), lacking antibiotic prescriptions for at least one year. The provided fecal samples were plated for the presence of *E. coli* and other enterobacteria resistant to trimethoprim/sulfamethoxazole (TMP/STX), ciprofloxacin (CIP) and 3^rd^ generation cephalosporins (3GC). *E. coli* was also characterized as belonging to the pandemic multi-drug resistant clonal groups ST131 (subclone H30) and ST1193. Following sample collection, the women were monitored for 18 months for occurrence of UTI.

*E. coli* was cultured from 90.8% fecal samples, with 24.1% containing bacteria resistant to TMP/STX, 19.4% to CIP, and 7.9% to 3GC. In 62.5% samples, only all-susceptible *E. coli* were present. Overall, there were no age-related differences in resistance prevalence. However, while the total *E. coli* H30 and ST1193 carriage rates were similar (4.3% and 4.2%, respectively), there was a notable increase of H30 carriage with age (P = .001), while carriage decreased with age for ST1193 (P = .057).

Within 18 months, 184 women (10.2%) experienced at least one episode of UTI - 10.9% among the gut *E. coli* carriers and 3.0% among the non-carriers (P=.0013). The UTI risk among carriers of *E. coli* H30 but not ST1193 was significantly above average (24.3%, P = .0004). The UTI probability increased with age, occurring in 6.4% of 50–59 yo and 19.7% of 80+ yo (P<.001), with the latter group being especially at high risk for UTI, if they were colonized by *E. coli* H30 (40.0%, P<.001).

*E. coli* was identified in 88.1% of urine samples, with 16.1% resistant to TMP/STX, 16.1% to CIP, 4.2% to 3GC and 73.1% to none of the antibiotics. Among tested urinary *E. coli* resistant to antibiotics, 86.1% matched the resistance profile of *E. coli* in the fecal samples, with the clonotyping and whole genome sequencing confirming the matching strains’ identity. Positive predictive value (PPV) of using gut resistance profiles to predict UTI pathogens’ susceptibility to TMP/STX, CIP, 3GC and all three antibiotics were 98.4%, 98.3%, 96.6% and 95.3%, respectively. Corresponding negative predictive values (NPV) were 63.0%, 54.8%, 44.4% and 75.8%, respectively. The AUC ROC curve values for the accuracy of fecal diagnostic testing for the prediction of UTI resistance ranged .86-.89. The fecal test-guided drug-bug mismatch rate for empirical (pre-culture) prescription of TMP-SXT or CIP is reduced to ≤2% in 89.6% of patients and 94.8% of patients with an optional 3GC prescription.

**Conclusion.:**

The resistance profile and clonal identity of gut colonizing *E. coli*, along with the carrier’s age, can inform personalized prediction of a patients’ UTI risk and the UTI pathogen’s antibiotic susceptibility within an 18-month period.

## Introduction

The personalized or precision medicine approach is based on defining the potential risks, expected outcomes, and optimal treatment of diseases based on an individual’s unique biomarkers, which are usually genetically determined but can also depend on and behavioral factors [1]. The indigenous microbiota that colonizes the gut, vagina or other body compartments is considered an integral component of the human organism, influencing various aspects of individuals’ health (for review see [2, 3]). At the same time, the resident microflora serves as a major source of opportunistic bacterial pathogens for the host, including those resistant to multiple antibiotics [4–6]. However, it remains unclear how the identification of certain bacterial species and/or sub-species lineages in the colonizing microbiota can predict the person’s risks for developing specific clinical infections or guide antibiotic treatment choices, especially in the era of continuously increasing antimicrobial resistance.

Gut resident microbiota, *Escherichia coli* and other enterobacterial species in particular, is the main reservoir for strains causing the most common bacterial infection treated by general practitioners - community-acquired urinary tract infections (UTIs) with the estimated total annual incidence rate of up to 7% the USA and globally [7–10]. Postmenopausal women, typically aged 50 or older, face a particularly high risk of developing severe and drug-resistant forms of UTIs [11, 12]. While acute uncomplicated cystitis is the most common form of UTI, its very high incidence places a substantial financial burden on the healthcare system, especially if the initial treatment choice was not effective [10, 13]. Also, more severe UTI forms such as recurrent cystitis, pyelonephritis, or urosepsis (an estimated one-third of sepsis cases begin as UTIs) significantly contribute to the overall costs associated with UTIs, particularly in elderly [10, 13, 14].

UTIs are almost exclusively treated with antibiotics, thus representing a primary single reason for antibiotic use in humans [15]. However, antimicrobial resistance has reached pandemic proportions in the last few decades, contributing to increased mortality rates and healthcare costs (estimated at approximately $2,000 per ineffective prescription) [16–19]. Because determining the resistance profiles of infecting bacteria takes 2–3 days, physicians must resort to the empirical (pre-culture) choice of antibiotics. Typically, empirical treatment is inherently imprecise as it is not informed by the specific resistance profile of infecting bacteria but relies on general resistance patterns in the local community [20]. For empiric treatment of uncomplicated UTI, the Infectious Diseases Society of America (IDSA) recommends treatment with an antibiotic if the local resistance prevalence in *E. coli* is no more than 20%, whereas for pyelonephritis or other severe forms, the recommended resistance prevalence should be ≤10% [20–22]. Unfortunately, the resistance to commonly used antibiotics is now often well above 20% and the empiric guesswork results in the prescription of ineffective antibiotics (‘drug-bug mismatches’) in 15–35% of cases, leading to increased health care cost and overuse of last-line, broad-spectrum antimicrobials [16, 18, 19].

It has been well-established that extraintestinal pathogenic *E. coli* and other enterobacteria causing UTIs derive from the gut, where they reside as part of the commensal microbiota, typically existing as a consortium of multiple strains (for review see [23]). These commensals include *E. coli* strains resistant to antibiotics, and their presence in the gut may not be contingent upon antibiotic use by the individual [24–28]. Among the antibiotic resistant *E. coli* causing UTIs, strains of two multi-drug resistant clonal groups (genetic families) dominate in the USA and globally – one is the sequence types ST131-H30R/Rx (aka ST131 clade C1/C2; *E. coli* H30 for further) and ST1193 [24, 25, 27, 29–33]. Their rampant spread was noted in the first and second decades of this century, respectively, with fluoroquinolone resistance being the signature of both clonal groups. It has been hypothesized that the high prevalence and global spread of *E. coli* H30 and ST1193 could be connected to their success as gut colonizers [25, 34–36].

Multiple studies have estimated the risks of extraintestinal infections associated with gut carriage of multi-drug resistant *E. coli*. Generally, these studies have focused on the risks of bacteremia and included samples from hospital/ICU-admitted patients and/or those who underwent various organ transplant procedures [37–40]. The correlation between gut carriage and consequent drug-resistant infections has been sufficiently strong to motivate studies on gut decolonization of patients at risk, employing methods such as fecal microbiome transplantation, probiotics, and/or phage therapies [37, 41–44]. To our knowledge, however, there have been no observational prospective studies to assess the value of characterizing gut-colonizing bacteria for predicting the risks and guiding the antimicrobial treatment of such a vastly common antibiotic-treated medical condition as UTI. Additionally, while it is well-known that certain risk groups for UTI exist, such as patients with diabetes, postmenopausal women or, the elderly [45–47], it is unclear to what extent the probability of UTI in those groups are also defined by the species or clonal type of gut-colonizing enterobacteria.

Here, we present the outcomes of an 18-month prospective observational study on the risk for UTI women above 50 yo and the correlation between the clonal and resistance profiles of *E. coli* in pre-collected fecal samples and those isolated from the follow-up clinical urine samples. The study group included 1,804 women receiving out-patient care in the Kaiser Permanente system in Washington State, USA (KPWA), serving mostly the metropolitan Seattle/Puget Sound Area, who had not received any antibiotics for at least one year before providing the fecal sample. We demonstrate that the UTI risk, while dependent on age, varied considerably based on the clonal identity of gut-colonizing *E. coli*. Additionally, determining the resistance profile of gut *E. coli* significantly improves the accuracy of resistance prediction for UTI-causing strains against three commonly used antibiotics: trimethoprim/sulfamethoxazole (TMP/STX), ciprofloxacin (CIP), and 3^rd^ generation cephalosporins (3GC). Thus, we believe that the analysis of gut microbiota for the presence, resistance profile, and clonal identity of resident *E. coli* shows potential for a personalized medicine approach in the clinical management of UTI, empowering antimicrobial stewardship efforts and, potentially, developing effective means for infection prevention.

## Methods

### Study design and participants.

This prospective cohort study from May 1, 2021 through December 31, 2021 was conducted among women aged 50 years and older enrolled in Kaiser Permanente Washington (KPWA). One-year membership prior to enrollment was required to clinical and demographic data prior to baseline. Besides enrollment, age and gender, exclusion criteria included any antibiotic prescription, UTI and placement in a long-term care facility within a year prior to enrollment in the study. This study was approved by the KPWA) Research Institute Institutional Review Board. The procedural details pertaining to the mailing of study kits to participants are described in the previous manuscript [24].

***Processing of fecal samples and typing of fecal E. coli*** was described in detail previously [24]. Briefly, fecal samples were plated on either pre-poured HardyCHROM™ UTI agar plates (Hardy Diagnostic, USA) or plates prepared from HiChromeTM UTI Agar (HiMedia Laboratories Pvt, Ltd.) supplemented with ciprofloxacin (CIP, 0.5 mg/L), trimethoprim/sulfamethoxazole (TS, 4/76 mg/L), ceftazidime (CAZ, 8 mg/L), ceftriaxone (CTX, 2 mg/L). Plates were incubated 16-20h at 37° C, and single colonies (SCs) morphologically identified as potential *E. coli* were cultured, saved and tested further for (a) resistance to CIP, TMP/STX and 3^rd^ generation cephalosporins (3GC – CAZ and CTX), (b) clonality based on sequencing of four loci – *fumC*, *fimH*, *gyrA* and *parC*, as described previously [24].

### Processing of clinical urine samples and typing of urine E. coli.

If a study participant submitted a urine sample to KPWA clinical laboratory, a routine urinalysis test was performed, followed by culture and sensitivity testing if required. Starting February 2022, the UW laboratory was provided an aliquot of the urine sample, which was processed using the same protocol as fecal sample described above, adding *E. coli* clonality where applicable to sample information.

### UTI identification.

The incidence of urinary tract infections in study participants was deduced from their electronic health records (EHRs). Records were examined once all participants had surpassed 18 months following the submission of the fecal sample. UTI was characterized based on the criteria outlined in Bruxvoort et al [7], including UTI diagnosis codes, antibiotic prescriptions, urinalysis test results, and culture findings.

### Resistant E. coli isolates comparison using whole genome sequencing.

Whole genome sequencing was conducted using the Illumina MiSeq platform following the manufacturer’s guidelines. Genomic DNA libraries were prepared using the Nextera XT Library Prep Kit (Illumina, CA). Raw data were uploaded to the Enterobase database (https://enterobase.warwick.ac.uk/) for genome assembly and allele calling processes. Core gene sequences were utilized to assess the phylogenetic relationship among isolates within the same clones.

### Search for sensitive E. coli clones within fecal samples.

Fecal samples were examined to identify the presence of the *E. coli* clone responsible for UTI in the participant. Three methods were employed. Firstly, a minimum of two individual single colonies were isolated directly from the sample during initial processing. These colonies were then screened for resistance profiles and clonal identity. If these isolated colonies did not match the uropathogenic *E. coli* clone, further isolation and screening of 9–28 (averaging 13) individual colonies were conducted. Secondly, the uropathogenic *E. coli* strains were subjected to testing for resistance against a broader spectrum of antibiotics, including ampicillin (50 and 100 mg/L), rifampin (2 and 4 mg/L), tetracycline (16 mg/L), cefazoline (4 and 8 mg/L), chloramphenicol (16 and 32 mg/L), kanamycin (12.5 and 25 mg/L), gentamicin (15 mg/L), spectinomycin (15 mg/L), streptomycin (50 mg/L), trimethoprim (8 and 16 mg/L), and sulfamethoxazole (80 and 160 mg/L). Subsequently, fecal samples were plated on the antibiotic of interest to search for isolates exhibiting the same resistance profile. Thirdly, fecal samples were resuspended in phosphate-buffered saline (PBS) and between 10^3^ −10^4^ cfu/plate were cultured on McConkey agar. Plates were divided into sectors, and bacteria from each sector were collected and re-suspended in 1 mL PBS. A 100 uL aliquot of this suspension was used for DNA purification using 5% Chelex 100 (Bio-Rad, Richmond, CA) [48]. The purified DNA was then subjected to qPCR to detect the presence of urinary clone-specific loci and/or SNPs. In the event of a positive reaction, the saved bacterial suspension was plated again to obtain and test individual single colonies.

### Whole genome sequencing.

Matching sets of *E. coli isolates* from clinical urine samples and predating fecal samples underwent sequencing on Illumina MiSeq platform using MiSeq 600 cycle v3 kit (Illumina). Raw reads were uploaded to Enterobase database (https://enterobase.warwick.ac.uk/) where they were assembled. The core genes were then downloaded from Enterobase and analyzed for sequence identity. The trees were bult using MEGA v7 software. Enterobase uberstrain names are listed in Table S3.

### Statistical analysis.

Chi-square test was used to evaluate 2×2 comparisons. Logistic regression was used to analyze the prevalence of different *E. coli* strains and the incidence of UTI among participants across different age groups. The analysis was conducted using STATA 14.2 Software (StataCorp, Texas, USA).

## Results

### Antibiotic resistant E. coli are carried at high rates by women without recent exposure to antibiotics.

We collected fecal samples from 1,804 women of age 50 years or above who did not take antibiotics for at least one year prior to enrollment. Among the enrollees, 437 women (24%) were of age 50–59, 632 (35%) aged 60–69, 532 (30%) aged 70–79 and 203 (11%) aged 80 and above (Table 1) (Fig S1A).

As expected, *E. coli* was present in a vast majority of the fecal samples (90.8% on average) across all age groups, with a slight but statistically significant decreased *E. coli* carriage observed for younger enrollees (Table 1) (Fig S1B). The *E. coli* positive samples were tested for the presence of any resistance to TMP/STX, CIP and 3GC. In 62.5% samples, all *E. coli* were sensitive to all three antibiotics, 24.1% samples contained *E. coli* resistant to TMP/STX: 19.4% to CIP and 7.9% to 3GC (Table 1), with 10.8% having *E. coli* resistant to more than one antibiotic. There were no differences between the age groups in prevalence of samples with resistant *E. coli* (Table 1) (Fig S1C–E).

Among the *E. coli*-positive fecal samples, clonal groups *E. coli* H30 and ST1193 were found among 4.3% and 4.2%, respectively, with a combined prevalence of 43.4% among the samples with CIP-resistant *E. coli*. The gut carriage of *E. coli* H30 significantly increased with age (P = .001), ranging from 2.3% in the 50–59 yo group to 7.9% in women of 80+ yo (Table 1) (Fig S1F), while an opposite age-dependence tendency was observed for the carriage of ST1193, with 5.2% in the youngest group but only 2.1% (P = .057) in the elder group (Table 1) (Fig S1G).

Thus, a third (34.1%) of women 50 yo and older without recent exposure to antibiotics carry gut *E. coli* that is resistant to at least one of the three commonly used antibiotics, with no overall differences in the resistant *E. coli* carriage between different age groups. At the same time, the older women tended to carry significantly more frequently *E. coli* from the multi-drug resistant clonal group ST131-H30.

### The 18-month probability of UTI occurrence depends on E. coli presence and clonality.

During the follow up 18 months of observation, 5–17 enrollees per month were diagnosed with UTI (Fig S2), with a total 184 women (10.2%) having at least one episode of UTI (Table 2). All urines were collected from women as outpatients; thus, all UTI cases qualify as being community acquired. The frequency of UTI cases significantly increased with age (Fig S1H), occurring in 1 in 5 (19.7%) women of age 80+ (Table 2). Interestingly, across the age groups UTI occurred nearly four-fold more frequently among women who carried *E. coli* in the gut than in those with no *E. coli* presence in feces.

In the total cohort of enrollees, the 18-month rate of UTI did not significantly differ between the carriers of all-sensitive *E. coli* and those carrying *E. coli* resistant to any of the tested antibiotics (Table 2). In contrast, the rate of UTI was nearly significantly higher than average for the carriers of multi-drug resistant *E. coli* H30 (24.3%; p=.0004). Notably, the rate of UTI among the carriers of ST1193 was at the average level.

When analyzed by age groups, the rates of UTI increased with age among the carriers of all-sensitive, TMP/STX-resistant, and CIP-resistant *E. coli* but the increase with age did not reach the statistical difference across the 3GC carriers (Fig S1I–M). While the UTI rates did not change with age among the carriers of ST1193, the UTI rates increased with age among the *E. coli* H30 carriers, reaching the 18-month occurrence of 36.4% in women or age 70–79 and 40.0% of age 80+. Moreover, in the two elder groups of enrollees, the UTI probability for the *E. coli* H30 carriers was significantly higher than for carriers of any other *E. coli*.

Thus, nearly 1 in 8 of women aged 50 and above, who are not taking antibiotics but carry *E. coli* in their gut, would experience a UTI within an 18-month period. In the older women, however, the overall UTI frequency increases up to two-fold overall and nearly four-fold in those who are colonized with the multi-drug resistant *E. coli* H30.

### E. coli is the predominant and most resistant urinary pathogen.

To evaluate the identity and resistance profiles of urinary pathogens we analyzed a total of 134 bacteriology-positive urine samples submitted by our study enrollees to the clinical laboratory as part of the initial UTI diagnostics. The bacteriology-positive samples were evenly spread across the 18 months of observation (Fig S2). *E. coli* was identified in 118 (88.1%) of the samples and was predominant at a similarly high level across all ages. Out of the remaining 16 samples, more than half (9 samples) contained *Klebsiella* spp., with the rest having *Citrobacter* spp. (in 2 samples), *P. mirabilis* (3), *Salmonella enterica* (1) and *E. faecalis* (1). Among the urine-isolated *E. coli*, 82 (69.5%) isolates were sensitive to all three antibiotics, 19 (16.1%) were resistant to TMP/STX, 19 (16.1%) to CIP and 5 (4.2%) to 3GC (Table 3), with 6 (4.6%) resistant to multiple antibiotics (Table S1). Among the non- *E. coli* urinary pathogens, none were resistant to any of the tested antibiotics, which is significantly lower (p=.008) than the proportion of resistant *E. coli* among the clinical isolates (Table S2).

Thus, in the community-acquired UTI among the women aged 50 and above, *E. coli* is both the predominant pathogen and responsible for most of the antibiotic-resistant infections.

### Resistance profile of E. coli in the urine samples match those in fecal samples due to the strains’ identity.

Baseline fecal *E. coli* was available from all 118 enrollees with *E. coli* positive UTIs. Among the fecal samples, 64.2% contained all-sensitive gut *E. coli*, 20.3% contained *E. coli* resistant to TMP/STX, 23.3% to CIP and 6.8% to 3GC. There was a highly significant (P < .001) overlap between the presence of resistant *E. coli* in urine and the corresponding fecal samples – out of 36 urine isolates resistant to any of the three antibiotics, 31 (86.1%) had an *E. coli* with the same resistance pattern in the corresponding fecal samples. Among them, 89.5% of urine isolates had an *E. coli* with a matching resistance to either TMP/STX or FQ and 80% for the resistance to 3GC (Table 3). Similarly, in 83.7% cases when the urine *E. coli* was sensitive to all three antibiotics, there was no resistant *E. coli* in the corresponding fecal sample.

The high-level correspondence between the resistance phenotypes of fecal and urinary samples obtained within 18 months of each other suggests that the same *E. coli* strain is present in both sample types. Indeed, in 21 cases when both fecal and urine sample were available for genetic clonotyping and contained isolates resistant to the same antibiotic(s), all of them were of the same clonal sub-group (Table S1). We compared 9 pairs of such clonally matching strains by whole genome sequencing and confirmed that the same-person urine and fecal samples are much closer genetically to one another than other strain pairs even of the same clonotype (Fig S3).

Five sample pairs with mismatched resistance profiles between fecal and UTI isolates were evenly spread across the 18 months of observation, obtained at months 4, 6, 13, 16 and 17 (Table S1). Out of those, 3 samples were available for the clonal identity, with all determined to be of a different nature.

Thus, there is a high correspondence between the resistance profiles of gut resident *E. coli* and UTI isolates obtained from the same person within 18 months from fecal sample.

### Urinary E. coli prediction based on fecal E. coli antibiotic resistance (FECAR) testing.

Because only a small portion of *E. coli* from urine samples did not have a fecal *E. coli* with a matching resistance profile, positive predictive values (PPV) for the susceptibility to antibiotics ranged between 94.5% for the all-sensitive urine isolates to 99.1% for those sensitive to 3GC (Table 4). The negative predictive values (NPV; prediction of non-susceptibility) ranged between 71.1% for any of the three antibiotics and 44.4% for 3GC. Notably, PPV and NPV remained at similar levels for the resistance prediction for the entire set of 134 urinary pathogens (i.e. including the 16 urine samples with non- *E. coli* bacteria, all of which as indicated above, were sensitive to the three antibiotics), because vast majority of the corresponding fecal samples had only all-sensitive *E. coli* (Table 4; Table S2). We estimated the diagnostic value of a potential fecal *E. coli* antibiotic resistance (FECAR) test to determine the resistance of UTI-causing pathogens in our cohort. The values of AUC (Area Under the Receiver Operating Characteristic (ROC) Curve) for the FECAR test ranged from .86-.89, i.e., at the ‘excellent’ level [49].

We analyzed how, hypothetically, the FECAR testing could improve the accuracy of empirical (pre-culture) treatment of the UTI cases in our study population by guiding the antibiotics choice. Using FECAR testing, TMP/STX, CIP and 3GC would be ‘*recommended’* for use in nearly 78%, 76% and 93% of all UTI cases, respectively, based on the absence of resistant *E. coli* in the corresponding fecal samples (Table 5). The FECAR-guided antimicrobial choice would lead to a drug-bug mismatch in only 1.9%, 2.0% and 0.8% of cases, respectively. Either TMP/STX or CIP would be recommended in almost 90% case and at least one of the antibiotics in 95% cases (Table 5).

Based on the 2022 annual antibiogram of *E. coli* isolated in the clinical microbiology laboratory of all KPWA enrollees, the local resistance to all three antibiotics is ≤20% - 16.9% to TMP/STX, 12.8% to CIP, and 5.8% to 3GCR. Thus, any of them can be recommended for the UTI treatment in our enrollees, assuming all patients had an uncomplicated cystitis. In our cohort, however, such FECAR-unguided treatment would lead to a drug-bug mismatch in 14.2% for TMP/STX and CIP, which is significantly higher than the mismatch resulted from the FECAR guidance (P = .0009 and P = .0011, respectively). For the unguided 3GC treatment the mismatch would occur in 3.7% of cases, which is not significantly higher (P = .0963).

In contrast, the use of TMP/STX, CIP and 3GC would be *‘not recommended*’ by FECAR in 21.6%, 23.9% and 6.8%, respectively, due to the presence of resistant *E. coli* in feces. While in 41.4, 46.9 and 55.6%, respectively, of the corresponding UTI cases, the isolate happened to be susceptible to the antibiotic, the antibiotics use in the not recommended cases would result in drug-bug mismatch rate that is significantly above the mismatch rates resulted from the unguided treatment, well exceeding the 20% rates considered to be acceptable by the IDSA recommendations.

## Discussion

Our study demonstrates that the presence and clonal identity of gut *E. coli* contribute to the risk of UTI. Moreover, although the human gut is often colonized by multiple strains of *E. coli* simultaneously (as discussed in detail below), the presence/absence of gut *E. coli* resistant to commonly used antibiotics could be used for predictive diagnostics of urinary pathogen susceptibility, within 18 months of the fecal testing and with an ‘excellent level’ of test accuracy. Thus, such testing can support a personalized medicine approach to UTI management by improving prediction of UTI infection and tailoring the empiric treatment choice.

Although the time of reaching menopause varies widely, women aged 50 and above are generally considered to be in the range of premenopausal to post-menopausal. Due to estrogen decline, a rise in vaginal pH, higher urine retention in the bladder, and other factors, postmenopausal women are known to be at an increased risk for severe, complicated, and drug-resistant forms of UTI [50, 51]. All women selected for the study did not have exposure to antibiotics and were not diagnosed with UTI or hospitalized for any reason at least one year prior to the study enrollment. Although these criteria may result in a study population with lower risk compared to a general population women 50 years and older, this would rather result in an underestimate of the potential impact of FECAR, which could be expected to have an even higher impact in a population with more frequent infection and therefore higher probability of resistance.

As expected, *E. coli* was isolated from the fecal sample of most women. Though a clear minority, *E. coli* non-carriers were ~2-times more common among women younger than 70. This is a novel finding, the underlying reason for which remains unclear. Also novel is the follow-up observation that *E. coli* non-carriers are about three times less likely to have UTIs than carriers, strongly suggesting that *E. coli* is the major causative species for UTI. These data provide further support that the probability of UTI depends on and, thus, may be predicted by the presence or absence of gut-colonizing *E. coli*.

We concentrated here on three antibiotics commonly used for the CA-UTI treatment. TMP/SMX (Bactrim) is suggested as the first line antibiotic for uncomplicated UTI, similar to nitrofurantoin or fosfomycin [52]. CIP is suggested as an alternative drug of choice when the first line antibiotics cannot be used for any reason, or as the first-choice antibiotic if pyelonephritis is suspected, usually combined with a single-dose boost of parenteral 3GC. However, as mentioned in the Introduction, the empirical use of antibiotics is recommended for the treatment of cystitis only if the local resistance prevalence is below 20%, or below 10% for the treatment of pyelonephritis [21].

Surprisingly, even though study participants did not take antibiotics for at least a year prior enrollment, just over a third of them carried gut *E. coli* resistant to one or more of the three antibiotics. Interestingly, the carriage of resistant *E. coli* did not differ significantly between age groups, in accordance with a lack of evidence that increased age is a risk factor for carriage of drug-resistant bacteria [28]. However, the lack of association could because our study enrolled only women who had not taken antibiotics in the year prior.

The most striking difference between the age groups was found in the carriage of *E. coli* belonging to the pandemic multi-drug resistant clonal groups *E. coli* H30 and ST1193. *E. coli* H30 is estimated to have emerged in the 1990s gained notoriety as the main cause of antibiotic resistant UTIs globally since early 2000s [33, 53]. Independently of resistance, it is also associated with complicated and severe forms of UTIs, such as recurrent cystitis, pyelonephritis, urosepsis, and infections in diabetic and immunocompromised patients [54]. ST1193 emerged later than *E. coli* H30 but started to challenge the latter in global UTI predominance in the mid-late 2010s [29, 31]. It has been proposed that the phenomenal global spread of both *E. coli* H30 and ST1193 is associated with their success as gut colonizers, possibly connected to the mutations responsible for CIP resistance [35, 36]. The fact that *E. coli* H30 is significantly more commonly carried by older women corresponds to our previous findings in a smaller prior study [11].

The underlying reason for the age differences in *E. coli* H30 and ST1193 carriage is not yet known. However, we believe that this observation suggests that resistance *per se* might not be the main selective factor for gut colonization and other host factors, e.g., immune status, may be in play. Whatever the underlying reason for the different carriage rates it’s important for predicting the occurrence of UTI. The overall 18-month incidence of UTIs in *E. coli* H30 carriers was twice as high as the average – 1 in 5 women. This was due to an astoundingly high rate of *E. coli* H30 UTIs in women aged 70+, occurring in more than a third of the enrollees, higher than in almost every other category of *E. coli* carriers of this age. Interestingly, the UTI rates for ST1193 carriers were not different from the average, potentially reflecting differences either in the urovirulence potential of the strains or in the host defense status of the colonized populations.

The significantly higher rate of UTIs for *E. coli* H30 illustrates the importance of predicting UTI based on the clonal identity of the resident strain, in combination with considering host demographics. While we did not examine the UTI incidence for other major clonal groups of *E. coli*, this finding highlights the value of examining the microbiota composition beyond large taxonomic groups or even species (as mostly done by microbiome studies) but at the sub-species, clonal group level that we propose to term as a ‘clonobiome’ analysis [55]. As with most human pathogens, the *E. coli* species is clonal by nature, with the majority of strains belonging to a relatively limited number of genetically well-defined and closely related clonal groups (i.e., Sequence Types or STs) that can significantly differ from each other in virulence potential and/or antimicrobial resistance profiles [56, 57]. Thus, by defining the clonal diversity of the colonizing strains, the clonobiome approach allows us to assess more accurately the clinical risks and treatment options associated with the resident microbiota. To note, it was shown that the clonal identity of multiple gut-colonizing *E. coli* strains can be directly determined in fecal samples by deep sequencing of just two genetic loci – *fumC* and *fimH* (CH typing) [55].

While predicting the probability of UTI in certain risk groups could be of practical interest, we believe that, if a clinical infection develops, it is even more important to accurately predict the antibiotic susceptibility of the pathogen. We demonstrate here that, within up to 18 months of fecal sample analysis, the resistance of the gut resident *E. coli* – dubbed here as FECAR test - served as a valuable predictive tool for estimating the susceptibility of the UTI-causing bacteria for the three tested antibiotics. Though this conclusion is based on the limited testing and data we have for our population in the study, the significant improvement in the potential antibiotic choice is reflected by the ‘excellent’ discrimination level of FECAR for the resistance to TMP/STX, CIP and 3GC based on the AUC values between 0.8 and 0.9 [49]. Due to the relatively low overall resistance to third-generation cephalosporins (3GC), the reduction in drug-bug mismatch rates in the allowed cases was not as obvious as with TMP/STX and CIP, but the AUC value was also at the excellent level. Moreover, because *E. coli* was responsible for nearly 90% of CA-UTI cases, the diagnostic prediction was also excellent for all UTI cases overall. Thus, the accuracy of predicting resistance profiles of UTI-causing *E. coli* based on the fecal sample collected within 18 months is sufficiently high to warrant further studies to develop this approach as a predictive tool for antibiotic selection in UTI. The FECAR-guided empirical choice of antibiotics could lead to a several-fold reduction of the drug-bug mismatches relatively to the unguided treatment.

Naturally, the correlation between the resistance profiles of gut *E. coli* and that of urine *E. coli* suggests that the strains causing UTIs derived from the gut colonizers. Indeed, clonal identity was established between the vast majority of fecal and urine strains with matching resistance profiles when they were obtained from the same individual. In our study, strain clonality was determined by a highly discriminatory sub-ST genotyping method [58, 59], strongly implying the identity of the strains that was confirmed by whole genome sequencing of representative strain pairs.

Previous studies varied significantly in their estimates of the duration of gut colonization by specific *E. coli* strains – ranging from several days to a few years, with a possible existence of ‘transient’ colonizers with a short-lived gut residence span and long-term ‘resident’ colonizers with a much slower turnover [60]. Though our study did not analyze the duration of gut colonization per se, it suggests that uropathogenic antibiotic-resistant strains can reside in the gut for a relatively long time, making the 18-month predictability of the urinary pathogen feasible. This corresponds to previous studies suggesting that *E. coli* H30 and ST1193 strains may be well-adapted as colonizers [12, 34, 35, 61]. Moreover, it has been proposed that the genetic factors contributing to gut colonization could serve as evolutionary pre-adapted (coincidental) urovirulence factors [62, 63]. Finally, our results support the hypotheses that persistent gut colonization by antibiotic-resistant *E. coli* may not necessarily depend on an antibiotic use by the host [26].

The human gut is usually colonized by multiple strains of *E. coli* simultaneously. For example, it has been shown that *E. coli* H30 in the gut is found, on average, with 1–2 other strains, often exhibiting different resistance profiles and in different relative proportions. While in the current study, the genetic diversity of gut resident *E. coli* was not analyzed, colonization by multiple strains is expected for most enrollees. For example, the fact that in certain carriers of resistant strains, UTIs were caused by sensitive strains could be due, at least in some cases, to the possibility that resistant strains were co-colonizing the gut with a sensitive strain that eventually caused the infection. Alternatively, this could have happened because the resistant strain found originally in the fecal sample was replaced by the sensitive strain as part of the strains’ turnover. Gut strains dynamics could also explain the fact that, in a small number of cases, UTI was caused by a resistant strain not found in the fecal sample, which could be acquired by the host after the original sample collection. This, however, should be resolved in future studies.

It remains to be determined whether a more detailed exploration of gut *E. coli* strains’ diversity, fecal abundance, the presence/absence of putative virulence factors, and different resistance genes could further improve the prediction of the UTI risks and optimal treatment. In addition to validating our results, future studies should also assess whether FECAR could be used for prediction of the UTI pathogens resistance to a broader spectrum of antibiotics. Furthermore, the strain-dependent UTI risks need to be delineated across various age groups and genders, considering additional host factors such as co-morbidities, immunodeficiency status, etc. Despite the limitations of our study and remaining questions, we strongly believe that discerning gut *E. coli* profiles should become an integral component of the personalized medicine approach in UTI management. Additionally, our findings should prompt further research in the development of innovative strategies to prevent UTIs by, for example, selectively decolonizing gut carriers ofhighly urovirulent and/or multi-drug resistant enterobacterial strains. This may not only diminish risks for severe complications in the most vulnerable individuals but also contributes to curbing the antimicrobial resistance pandemic by reducing the transmission and community circulation of these pathogens.

## Supplementary Material

Supplement 1

## Figures and Tables

**Table 1.** Prevalence of different *E. coli* among women of different age

Characteristic ^a^	Total	Age		(yo)	P value ^b^	
	No. (%)	**50–59** **No. (%)**	**60–69** **No. (%)**	70–79 No. (%)	80+ No. (%)	
**Total**	1804 (100)	437 (100)	632 (100)	532 (100)	203 (100)	
***E. coli* present**	1638 (90.8)	388 (88.8)	561 (88.8)	500 (94)	189 (93.1)	.**001**
***E. coli* absent**	166 (9.2)	49 (11.2)	71 (11.2)	32 (6)	14 (6.9)	
**All-sensitive *E. coli***	1023 (62.5)	244 (62.9)	353 (62.9)	308 (61.6)	118 (62.4)	.583
**3GC-R *E. coli***	129 (7.9)	26 (6.7)	46 (8.2)	41 (8.2)	16 (8.5)	. 612
**TMP/STX-R *E. coli***	395 (24.1)	91 (23.5)	130 (23.2)	126 (25.2)	48 (25.4)	. 215
**CIP-R *E. coli***	318 (19.4)	74 (19.1)	113 (20.1)	97 (19.4)	34 (18)	. 655
** H30**	70 (4.3)	9 (2.3)	24 (4.3)	22 (4.4)	15 (7.9)	.**001**
** ST1193**	68 (4.2)	20 (5.2)	25 (4.5)	19 (3.8)	4 (2.1)	.057

a Prevalence of any or no *E. coli* carrying samples was calculated as percent from total number of samples; prevalence of resistant *E. coli* carrying samples was calculated as percent of any *E. coli* carrying samples.

b P values were derived from logistic regression using age in years as predictor and *E. coli* carriage as outcome, with sensitive/resistant *E. coli* carriage restricted for overall *E. coli* carriage in a sample; P < .05 in bold.

**Table 2.** UTI rates among different groups

Characteristic ^a^	Total	P value	Age (yo)				P value ^f^
	No. (%)		50–59 No. (%)	60–69 No. (%)	70–79 No. (%)	80+ No. (%)	
**Total**	184 (10.2)		28 (6.4)	51 (8.1)	65 (12.2)	40 (19.7)	<.**001**
***E. coli* present**	179 (10.9)	.**0013** ^b^	27 (7)	49 (8.7)	64 (12.8)	39 (20.6)	<.**001**
***E. coli* absent**	5 (3.0)		1 (2)	2 (2.8)	1 (3.1)	1 (7.1)	.796
**All-sensitive *E. coli***	121 (11.8)	.252 ^c^	22 (9)	33 (9.3)	45 (14.6)	22 (18.6)	.**001**
**3GC-R *E. coli***	12 (9.3)	.381 ^d^	1 (3.8)	4 (8.7)	4 (9.8)	3 (18.8)	.218
**TMP/STX-R *E. coli***	36 (9.1)	.131 ^d^	5 (5.5)	9 (6.9)	10 (7.9)	12 (25)	.**002**
**CIP-R *E. coli***	43 (13.5)	.449 ^d^	4 (5.4)	13 (11.5)	14 (14.4)	12 (35.3)	<.**001**
**H30**	17 (24.3)	.**0003** ^e^	0 (0)	3 (12.5)	8 (36.4)	6 (40.0)	<.**001**
**ST1193**	8 (11.8)	.821 ^e^	2 (10)	4 (16)	2 (10.5)	0 (0)	.927

a UTI rates are calculated as percentage of patients who had UTI from total number of females in this group.

b P value calculated in Chi-square test comparing UTI in *E. coli* carriers vs non-carriers; P < .05 in bold.

c P value calculated in Chi-square test comparing UTI in any resistant vs all sensitive *E. coli* carriers.

d P value calculated in Chi-square test comparing UTI in carriers of *E. coli* resistant to particular group of antibiotics vs all sensitive *E. coli*.

e P value calculated in Chi-square test comparing UTI in H30 or ST1193 vs any other *E. coli* carriers; P < .05 in bold.

f P values calculated in logistic regression with age as a predictor, unconstrained or constrained by group predictor; P < .05 in bold

**Table 3.** Coincidence of sensitive and resistant *E. coli* in clinical urine samples with identified uropathogen (N = 134) and respective fecal samples.

Characteristic	UTI	*E. coli* in Fecal sample	Overlap	P value ^a^
	No. (%)		No. (% UTI)	
		No. (% UTI)		
**Total UTI with identified uropathogen**	134 (100)			
**UTI caused by *E. coli*** (% total)	118 (88.1)	118 (100)	118 (100)	< .001
**All-sensitive *E. coli*** (% *E. coli* UTI)	82 (69.5)	73 (54.9)	69 (84.1)	< .001
**CIP-R *E. coli*** (% *E. coli* UTI)	19 (16.1)	31 (23.3)	17 (89.5)	< .001
**TMP/STX-R *E. coli*** (% *E. coli* UTI)	19 (16.1)	27 (20.3)	17 (89.5)	< .001
**3GC-R *E. coli*** (% *E. coli* UTI)	5 (4.2)	9 (6.8)	4 (80)	< .001
**UTI caused by non*- E. coli*** (% total)	16 (11.9)	15 (93.8)	N/A ^b^	
**All-sensitive non*- E. coli*** (% of non*- E. coli* uropathogen)	16 (100)	12 (75)	12 (75)	< .001

a P value calculated in logistic regression of the outcome (uropathogen) from predictor (fecal *E. coli*)

b N/A, not applicable

**Table 4.** Urinary *E. coli* prediction based on fecal *E. coli* sensitivity test results.

*E. coli in sample*	*E. coli* UTI (N = 118)			All known bug UTI (N = 134)		
	PPV, % [CI95]	NPV, % [CI95]	AUC ^a^	PPV, % [CI95]	NPV, % [CI95]	AUC ^a^
**All sensitive *E. coli***	94.5	71.1	0.8577	95.3	66.7	0.8577
	[86.6, 98.5]	[55.7, 83.6]		[88.5, 98.7]	[51.6, 79.6]	
**CIP-S *E. coli***	97.7	54.8	0.8767	98.0	53.1	0.8822
	[91.9, 99.7]	[36.0, 72.7]		[93.1, 99.7]	[34.7, 70.9]	
**TMP/STX-S *E. coli***	97.8	63.0	0.8894	98.1	58.6	0.8908
	[92.3, 99.7]	[42.4, 80.6]		[93.2, 99.7]	[38.9, 76.5]	
**3GC-S *E. coli***	99.1	44.4	0.8779	99.2	44.4	0.8806
	[95.0, 100.0]	[13.7, 78.8]		[95.6, 100.0]	[13.7, 78.8]	

a AUC, Receiving Operator Curve analysis (ROC) Area Under Curve

**Table 5.** Predictive power of knowing fecal bacterial resistance for treatment of UTI.

Antibiotic	Allowed		Resistance in allowed		Resistance overall		P value
	No.	%	No.	%	No.	%	
**TMP/STX**	105	78.4	2	1.9	19	14.2	.0009
**CIP**	102	76.1	2	2.0	19	14.2	.0011
**3GC**	125	93.2	1	0.8	5	3.7	.0963
**TMP/STX or CIP**	120	89.6	N/A	N/A	N/A	N/A	N/A
**TMP/STX or 3GC**	127	94.8	N/A	N/A	N/A	N/A	N/A
**CIP or 3GC**	126	94.0	N/A	N/A	N/A	N/A	N/A
**TMP/STX or CIP or 3GC**	127	94.8	N/A	N/A	N/A	N/A	N/A

N/A, not applicable
