# Supplementary material for: Gut resident Escherichia coli profile predicts the eighteen-month probability and antimicrobial susceptibility of urinary tract infections
Source: medRxiv. 2024 Apr 9:2024.04.05.24305377. Preprint. [Version 1] doi: 10.1101/2024.04.05.24305377 (PMC11030298; doi:10.1101/2024.04.05.24305377)
Supplement: Supplement 1 [file NIHPP2024.04.05.24305377v1-supplement-1.pdf]

medRxiv preprint doi: https://doi.org/10.1101/2024.04.05.24305377; this version posted April 9, 2024. The copyright holder for this preprint (which was not certified by peer review) is the author/funder, who has granted medRxiv a license to display the preprint in perpetuity.
It is made available under a CC-BY 4.0 International license .

Supporting information

S1 Fig. Analysis of gut *E. coli* carriage and UTI incidence by age.

S2 Fig. UTI incidence rates and uropathogens characteristics.

S3 Fig. Phylogenetic relationship between fecal (F) and clinical urine (CU) *E. coli* belonging to the same clonal group but isolated from different patients.

S1 Table. UTI *E. coli* resistant to antibiotics.

S2 Table. Non- *E. coli* UTI uropathogens.

S3 Table. All data by enrollee.
